# Elevated expression of HIGD1A drives hepatocellular carcinoma progression by regulating polyamine metabolism through c-Myc–ODC1 nexus

**DOI:** 10.1186/s40170-024-00334-6

**Published:** 2024-02-23

**Authors:** Haixing Zhang, Xiaoran Li, Ziying Liu, Zimo Lin, Kuiyuan Huang, Yiran Wang, Yu Chen, Leyi Liao, Leyuan Wu, Zhanglian Xie, Jinlin Hou, Xiaoyong Zhang, Hongyan Liu

**Affiliations:** 1 State Key Laboratory of Organ Failure Research, Guangdong Provincial Key Laboratory of Viral Hepatitis Research, Department of Infectious Diseases, Nanfang Hospital, Southern Medical University, Guangzhou, China; 2 Division of Hepatobiliopancreatic Surgery, Department of General Surgery, Nanfang Hospital, Southern Medical University, Guangzhou, China

**Keywords:** Hepatocellular carcinoma (HCC), Hypoxia-induced gene domain protein-1a (HIGD1A), c-Myc, Ornithine decarboxylase 1 (ODC1), Polyamine metabolism

## Abstract

**Background:**

Hypoxia contributes to cancer progression through various molecular mechanisms and hepatocellular carcinoma (HCC) is one of the most hypoxic malignancies. Hypoxia-inducible gene domain protein-1a (HIGD1A) is typically induced *via* epigenetic regulation and promotes tumor cell survival during hypoxia. However, the role of HIGD1A in HCC remains unknown.

**Methods:**

HIGD1A expression was determined in 24 pairs of human HCC samples and para-tumorous tissues. Loss-of-function experiments were conducted both *in vivo* and *in vitro* to explore the role of HIGD1A in HCC proliferation and metastasis.

**Results:**

Increased HIGD1A expression was found in HCC tissues and cell lines, which was induced by hypoxia or low-glucose condition. Moreover, HIGD1A knockdown in HCC cells arrested the cell cycle at the G2/M phase and promoted hypoxia-induced cell apoptosis, resulting in great inhibition of cell proliferation, migration, and invasion, as well as tumor xenograft formation. Interestingly, these anti-tumor effects were not observed in normal hepatocyte cell line L02. Further, HIGD1A knockdown suppressed the expression of ornithine decarboxylase 1 (ODC1), a rate-limiting enzyme of polyamine metabolism under c-Myc regulation. HIGD1A was found to bind with the c-Myc promoter region, and its knockdown decreased the levels of polyamine metabolites. Consistently, the inhibitory effect on HCC phenotype by HIGD1A silencing could be reversed by overexpression of c-Myc or supplementation of polyamines.

**Conclusions:**

Our results demonstrated that HIGD1A activated c-Myc–ODC1 nexus to regulate polyamine synthesis and to promote HCC survival and malignant phenotype, implying that HIGD1A might represent a novel therapeutic target for HCC.

**Supplementary Information:**

The online version contains supplementary material available at 10.1186/s40170-024-00334-6.

## Background

Hepatocellular carcinoma (HCC), a major type of liver cancer, ranks fourth in global incidence and third in mortality among malignant tumors [1]. In the past decade, despite notable advancements in novel drugs and therapies within the field of liver cancer treatment, along with progress in concepts such as early screening, integrated prevention, and collaboration treatment, the overall prognosis for liver cancer remains dismal [2]. The 5-year overall survival rate is only 12.1%, with merely 30% of patients having the opportunity for surgical resection [3]. Previous studies have indicated that tumor recurrence, metastasis, and drug resistance were the major causes of poor prognosis in HCC patients [4]. Therefore, further exploring the molecular mechanisms that participate in HCC development and progression would be useful for identifying novel drug targets.

HCC is among the most hypoxic malignancies, particularly within areas of tumor necrosis [5]. Moreover, treatments for HCC, such as transarterial chemoembolization that restricts blood supply to inhibit tumor growth, or tyrosine kinase inhibitors that reduce angiogenesis by inhibiting various kinase targets, can further aggravate intratumoral hypoxia [6–8]. Many studies revealed that within HCC tissues, the expression levels of hypoxia-inducible factors (such as HIF-1α, HIF-2α) or their target genes (GLUT1, LDHA, CA9, SLC7A1) are significantly elevated in comparison to non-tumorous liver tissue. Moreover, this elevated expression was linked to tumor recurrence, metastasis, and reduced patient survival rates [9, 10]. Functional research demonstrated that the signaling cascade of HIF molecules was involved in HCC cell proliferation, angiogenesis, invasion, and metastasis [11, 12]. Furthermore, it induced alterations in the glycolytic metabolic pathway, promoting tumor drug resistance [4]. Hence, the activation of hypoxia-related genes potentially played a crucial role in the malignant progression of HCC.

Hypoxia-inducible gene domain protein-1a (HIGD1A) is a HIF-1α targeted mitochondrial protein [13] which is typically induced *via* epigenetic regulation under hypoxia and low-glucose conditions [14]. HIGD1A mediates the assembly of cytochrome oxidase and the formation of respiratory chain complex [15]. During glucose starvation, HIGD1A reduced mitochondrial respiration, reactive oxygen species (ROS) production, and AMP-dependent protein kinase (AMPK) activity to promote cancer cell survival [16]. Additionally, HIGD1A was found to be released from mitochondria, followed by nuclear translocation, in response to severe stress such as ischemia [13] and radio/chemo-induced DNA damage [17]. Recently we found that HIGD1A was able to regulate hepatitis B virus transcription and replication [18], which is a major risk factor in the development of HCC. Interestingly, HIGD1A might play different roles in multiple types of cancer, such as tumor-promoting role in pancreatic cancer [19] and tumor-suppressing function in colon adenocarcinoma [20]. However, the biological effects of HIGD1A in HCC remain largely unknown.

In this study, we examined HIGD1A expression in HCC tissues and cell lines and explored the effect and mechanism of HIGD1A on the cancer phenotype and metabolism of HCC through *in vitro* and *in vivo* experiments. Our findings suggested that HCC exhibits an elevated expression of HIGD1A, potentially influencing polyamine metabolism by modulating the c-Myc pathway, thereby promoting the malignant phenotype of HCC.

## Methods

### Clinical samples collection

Paired tumor and adjacent non-tumor liver tissue samples were collected from 24 HCC patients during surgical procedures in the Nanfang Hospital of Southern Medical University (Guangdong, China) from 1st September 2019 to 1st February 2020. All specimens were snap-frozen in liquid nitrogen and stored at –80 ℃. Human tissues were collected with the required informed consent provided in writing by each patient and with the approval of the Medical Ethics Committee of Nanfang Hospital of Southern Medical University. This study was performed in accordance with the Declaration of Helsinki and the Declaration of Istanbul.

### Cell lines and cell culture

HepG2, Huh7, and MHCC97H cells were cultured in Dulbecco’s modified Eagle medium (DMEM) supplemented with 10% fetal bovine serum, 100 U/mL penicillin, and 0.1 mg/mL streptomycin (Gibco, USA), and L02 cells were cultured in RPMI1640 medium supplemented with 10% fetal bovine serum and antibodies. Cells were maintained at 37°C in a humidified atmosphere with 5% CO_2_. Hypoxia experiments were performed using a hypoxic chamber (Stemcell Technologies) with 1% O_2_ for 12 h. For glucose starvation, cells were incubated in glucose-free DMEM medium (Thermo Fisher Scientific) for 12 h. Polyamines rescue experiment was performed using 10 μM polyamines mix (Macklin) including the solution of putrescine, spermidine, and spermine as previously described [21]. During incubation with polyamines, 1 mM aminoguanidine was routinely added in the medium as an inhibitor of bovine serum amine oxidase to prevent oxidation of extracellular polyamines to toxic products.

### Lentiviruses and plasmids

The lentiviruses of shRNA-HIGD1A (shHIGD1A) for HIGD1A knockdown and shRNA-scramble (shCtrl) were purchased from GeneChem Biotechnology Co., Ltd. Lentiviral infection was performed following the manufacturer’s instruction. The infected cells were selected with puromycin (2 µM) treatment for 7–10 days. The pcDNA^TM3.1^-FLAG-HIGD1A plasmid expressing human HIGD1A (NM_001099669.2) and pcDNA^TM3.1^-FLAG-c-Myc plasmid expressing human c-Myc (NM_002467.6) were constructed by PCR from the respective cDNA clones using FLAG-tag-encoding oligonucleotides, followed by insertion into the pcDNA^TM3.1^ vector. The plasmid transfection was performed using Lipo3000 (Invitrogen, USA) according to the manufacturer’s instructions.

### Immunohistochemical (IHC) staining

IHC staining was performed as previously described [22]. Briefly, subcutaneous tumors were excised and fixed in formalin for 48 h. Serial 4-µm sections were cut, deparaffinized, blocked, and incubated at 4 °C overnight with the corresponding primary antibody: Ki67 (1:200, Cell Signaling Technology, 9449), c-Myc (1:200, Zen BioScience, 43250), ODC1 (1:200, Proteintech, 28728-1-AP). Then, an anti-rabbit/mouse IgG-HRP-linked secondary antibody (Genetech, GK500710) was added for 1 h at room temperature, followed by incubation with 3-diaminobenzidine tetrahydrochloride at room temperature for about 2 min. Hematoxylin was used to stain the nuclei. Finally, representative field photographs were captured using a BX63 microscope (Olympus) and analyzed using ImageJ software.

### Western blotting

Total proteins were isolated using RIPA lysis buffer (FD009, Fdbio science) and heated at 100 °C for 10 min. The protein from each group was separated by 12% SDS–PAGE and then transferred to polyvinylidene fluoride membranes (Roche, USA) for immunoblotting with the following primary antibodies: anti-HIGD1A antibody (Proteintech, 21749-1-AP), anti-HIF-1α antibody (BD Pharmingen, 610958), anti-DNMT1 antibody (Zen BioScience, R381634), anti-N-cadherin antibody (Affinity, AF4039-50), anti-E-cadherin antibody (Cell Signaling Technology, 3195T), anti-c-Myc antibody (Zen BioScience, 43250), anti-ODC1 (Proteintech, 28728-1-AP), anti-flag (Cell Signaling Technology, 2368S), anti-caspase3 (Cell Signaling Technology, 9662), anti-cleaved-caspase3 (Cell Signaling Technology, 9664), and anti-β-actin antibody (Cell Signaling Technology, 4970) at 1:1000 dilution. After blocking, the membrane was incubated with specific primary antibodies at 4 °C overnight. Subsequently, the membranes were probed with horseradish peroxidase-conjugated appropriate secondary antibody for 1 h at room temperature. Finally, the target bands were visualized using the ECL prime Western blotting detection reagent (GE Healthcare, USA) and detected with ImageQuant LAS 4000mini (GE Healthcare).

### RNA isolation and quantitative real-time PCR

Total RNA was extracted from samples using an EZ-press RNA Purification Kit (EZBioscience) according to the manufacturer’s instructions. Subsequently, reverse transcription reactions were performed using a Reverse Transcription System Kit (Accurate Biology), and real-time PCR was performed with SYBR qPCR Mix (Accurate Biology) on a Roche 480 real-time PCR machine (Roche). Relative expression was normalized to GAPDH by the 2^-ΔΔCT^ method. The primer sequences were listed in Supplementary Table 1.

### Cell proliferation assay

Cell proliferation was estimated using a Cell Counting Kit-8 (CCK8, Fude Biological Technology Co., China). In detail, cells were plated in 96-well plates at a density of 3000 cells per well and cultured in growth medium. Subsequently, CCK‐8 reagent (10 µL) was added to each well, and the cells were incubated for 1 h at 37 °C. Finally, the optical density was determined at 450 nm using Gen5 software (Biotek, USA).

### Cell cycle assay

For the cell cycle assay, cells were harvested, fixed in 70% ethanol, and stored at 4 °C overnight. Subsequently, after washing with phosphate-buffered saline (PBS), cells were resuspended in propidium iodide (PI) staining buffer containing RNase (BD Biosciences, USA) at room temperature for 15 min. DNA content was analyzed by flow cytometry using a FACS Canto^TM^ II FlowCytometer (BD Biosciences, USA). The percentages of cells in the various phases of the cell cycle were determined using ModFit LT 4.1 software (BD Biosciences). For aphidicolin (APH) induced cell cycle synchronization, HepG2, Huh7, and MHCC97H cells were pre-treated with 3 µg/ml APH for 24 h, washed with warm culture medium twice, and incubated in fresh culture medium for 24 h before being stained for cell cycle markers.

### Cell apoptosis assay

Cells were seeded in six-well plates overnight. After being cultured for 24 h, cells were collected, washed with PBS, and resuspended in a binding buffer containing Annexin V-APC and 7-AAD (BioLegend, USA) for 15 min at room temperature. After staining, the percentage of apoptotic cells was determined based on the populations of early and late apoptotic cells (Annexin V+) using a FACS Canto^TM^ II FlowCytometer (BD Biosciences, USA). To measure cell apoptosis induced by hypoxic condition, the cells were left for 12 h under hypoxic condition and then the apoptotic cells were detected with an Annexin V/7-AAD Apoptosis Detection Kit as previously described.

### Transwell migration and invasion assays

Cell migration and invasion ability were determined using Corning transwell insert chambers (Corning, USA) with and without Matrigel (Corning, 354248), respectively. For the cell migration assay, 4 × 10^4^ cells suspended in serum‐free DMEM were seeded into chambers whereas the lower chamber contained DMEM with 20% FBS as a chemoattractant. After 48 h, non-migrated cells were removed from the upper chambers, while cells that migrated through chambers were fixed using polyformaldehyde for 15 min. Then the fixed cells were stained with 1% crystal violet for 10 min. Following four washes with PBS, migrating/invading cells were observed, and images were captured using an inverted microscope (IX73, Olympus, Japan). For the cell invasion assay, the membrane was pre-coated with Matrigel to form a matrix barrier. Otherwise, the procedure was the same as that used for the migration assay.

### RNA-sequencing (RNA-seq) analysis

Total RNA was extracted from shCtrl- and shHIGD1A-infected HepG2 cells using a TRIzol reagent kit (Invitrogen, USA). After RNA quality control, sequencing libraries were constructed. Next, DEseq2 software was used to identify the differentially expressed genes (DEGs). The cut-off criteria for DEG selection were |log_2_ fold change| ≥ 1 and adjusted *P*-value <0.05. A list of DEGs is provided in Supplementary Table 2. Gene set enrichment analysis (GSEA) [23] was used to analyze the functional enrichment of DEGs. All GSEA results are available in Supplementary Table 3.

### Chromatin immunoprecipitation (ChIP) assay

This assay was performed using a chromatin immunoprecipitation kit purchased from Cell Signaling Technology (9003S) according to the manufacturer’s protocol. First, HepG2 and MHCC97H cells were transfected with the pcDNA^TM3.1^-FLAG-HIGD1A plasmid for 24 h as previously described. Then DNA and proteins were crosslinked with 1% formaldehyde and chromatin was digested using micrococcal nuclease. Then, the digested chromatin was incubated with 2 µg of anti-FLAG (DIA-AN, 2064) or anti-IgG antibodies at 4 °C overnight. The resulting antibody–chromatin complexes were incubated with 30 µl of protein G magnetic beads for 2 h. Bound DNA–protein complexes were eluted, and crosslinks were reversed after a series of washes. Purified DNA was resuspended in TE buffer (10 mM Tris-HCl, pH 8.0, 1 mM EDTA) for PCR analysis. Primers targeting the c-Myc promoter were listed in Supplementary Table 1.

### Dual-luciferase reporter assay

The promoter of c-Myc (−2000 to +160 bp) was subcloned and inserted into a pGL3-Basic vector (Promega). HepG2 and MHCC97H cells in a 24-well plate were transfected with the promoter reporter plasmid and vectors expressing the gene of interest using Lipofectamine 3000; pRL-TK was used as a transfection efficiency control. Cell lysates were harvested 24 h after transfection, and then firefly and Renilla luciferase activities were measured using a Dual-Luciferase Reporter Assay System (Promega). All reporter assays were repeated three times, and the data are shown as mean ± standard deviation.

### In vivo experiments

The Animal Ethics Committee of the Nanfang Hospital of Southern Medical University (China) approved all protocols for experimental animal studies. Male BALB/c-nu nude mice, aged 3–5 weeks, were acquired from Hunan SJA Laboratory Animal Co., Ltd., in China. The animals were kept in a specific-pathogen-free vivarium. Mice were randomly divided into two groups. HepG2, Huh7, and MHCC97H cells (5 × 10^6^ cells per mouse) carrying shCtrl or shHIGD1A constructs were mixed with 50 µl of PBS and then injected under the skin of nude mice on the side. After the tumors had become visible to the naked eye, they were measured every 3 days using a caliper. The volume of the tumors (V) was then calculated as V (mm^3^) = 0.5 × length × width^2^. After 18 days of implantation, the mice were euthanized, and their tumors were weighed, photographed, and then fixed in 4% paraformaldehyde overnight. For the orthotopic tumor model in the liver, shCtrl or shHIGD1A lentivirus transduced HepG2, Huh7, and MHCC97H cells (2 × 10^6^ cells per mouse) with 20 µl of PBS were intrahepatic-ally injected into the liver of BALB/c nude mice. Two weeks after cell injection, all mice were euthanized and the livers of mice were isolated, weighed, and photographed.

### Polyamines determination by high-performance liquid chromatography (HPLC)

Polyamines and amino acids were analyzed by LipidALL Technology as previously described [24]. Metabolites were extracted with methanol: water (8:2, v/v) at 4°C for 30 min at 1500 rpm [25]. At the end of the incubation, samples were centrifuged at 4°C for 5 min at 12,000 rpm. Clean supernatant was transferred to a new tube and dried in a SpeedVac under OH mode with no heating. The metabolite extract was reconstituted in 2% acetonitrile in water containing isotope-labeled standards for quantification of amino acids, and 1,6-diaminohexane (Sigma-Aldrich) was used for quantitation of polyamine. Polyamine and amino acids were analyzed on a Jasper HPLC system coupled to a Sciex 4500 MD. Metabolites were separated on a Waters Acquity UPLC HSS-T3 column (3 × 100 mm, 1.8 µm) using water containing 0.1% formic acid as mobile phase A and acetonitrile as mobile phase B.

### Statistical analysis

Software including IBM SPSS Statistics (version 26.0) and GraphPad Prism (version 8.0) were used to analyze the data. Methylation levels of the HIGD1A gene were analyzed using the UALCAN web tool (http://ualcan.path.uab.edu/). Statistical differences were determined by two-tailed t-tests in two-group comparisons. Analysis of variance was used to assess the statistical differences among several groups. A *p*-value level of less than 0.05 was deemed to indicate statistical significance.

## Results

### Elevated HIGD1A expression in HCC is induced by hypoxia or low-glucose condition

**Fig. 1:**
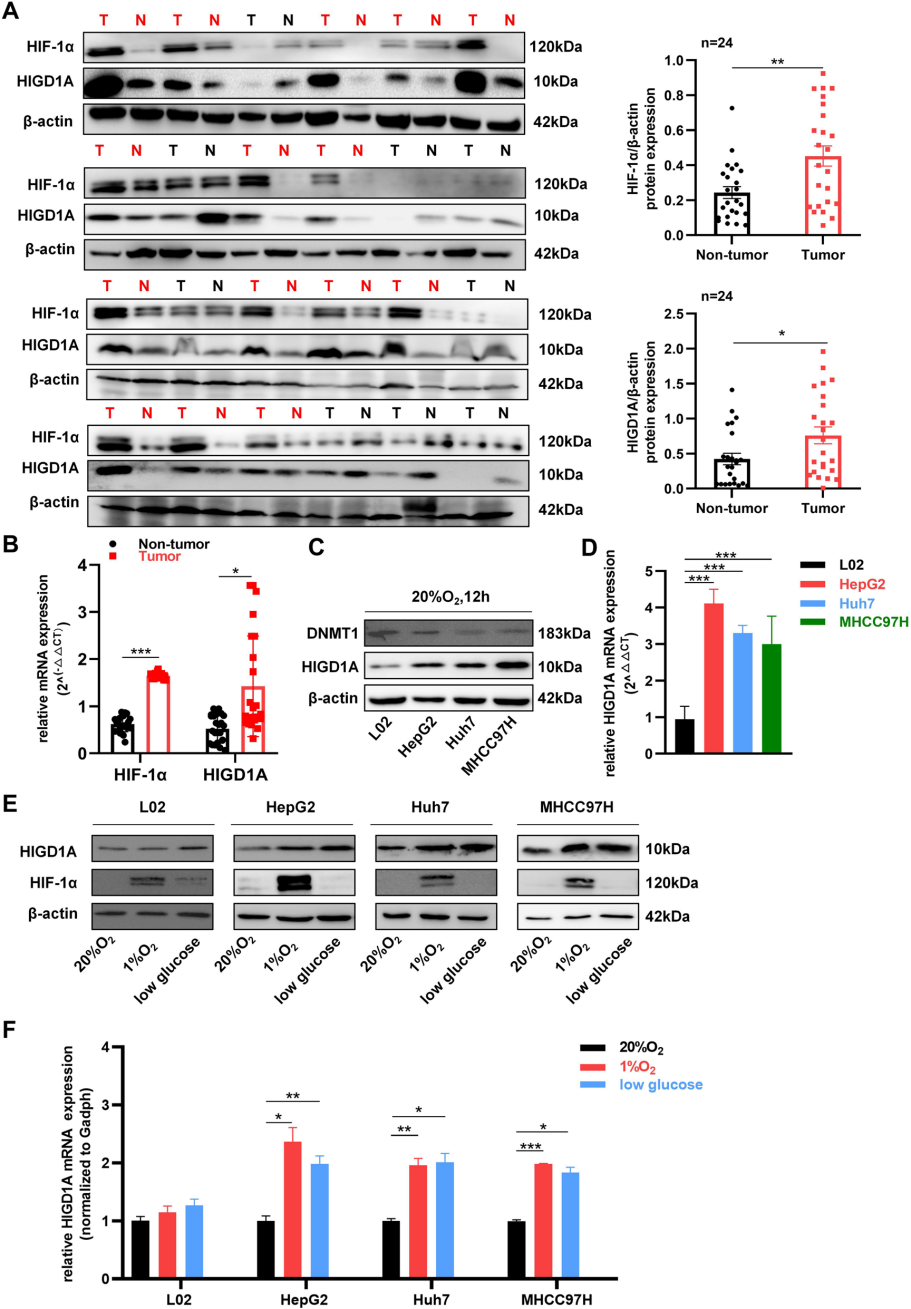
HIGD1A is overexpressed in HCC and is induced by hypoxia or low-glucose condition. **A** HIGD1A and HIF-1α protein levels in 24 pairs of HCC and adjacent normal tissues were analyzed by western blotting. The red color indicated pairs with increased HIGD1A expression in cancerous tissue. The grey scales of protein were analyzed by Image-Pro Plus software and were normalized by β-actin. T, tumor; N, non-tumor. **B** HIGD1A and HIF-1α mRNA levels in 24 pairs of HCC and adjacent normal tissues were analyzed by qRT-PCR. **C** Protein expression of HIGD1A and DNMT1 in L02, HepG2, Huh7, and MHCC97H cells. **D** Expression of HIGD1A mRNA in L02, HepG2, Huh7, and MHCC97H cells. **E** Western blotting analysis of HIGD1A protein expression in different cells cultured under 20% O_2_ or low glucose or 1% O_2_ for 12 h. **F** qRT-PCR analysis of HIGD1A mRNA expression in different cells cultured under 20% O_2_ or low glucose or 1% O_2_ for 12 h. Results were shown as mean ± SEM. *P <0.05, **P <0.01, ***P <0.001

First, HIGD1A expression was examined within a cohort of paired tumor tissues and adjacent non-tumor liver tissues from 24 HCC patients. The protein and mRNA levels were significantly higher in tumor samples when compared to adjacent normal liver tissues, as determined by western blotting and qRT-PCR (Fig. 1A and B). Given that HIF-1α exhibited regulatory effects on HIGD1A [26], the protein and mRNA levels of HIF-1α also showed a similar change trend as HIGD1A (Fig. 1A and B). In a consistent manner, HIGD1A expression in the HCC cell lines HepG2, Huh7, and MHCC97H, were higher than normal human hepatocyte L02 cells (Fig. 1C and D). Previous studies had shown that HIGD1A expression was regulated by DNA methyltransferase 1 (DNMT1) [27], a pivotal enzyme responsible for executing DNA methylation and epigenetic modulation. As expected, we confirmed that DNMT1 protein expression was lower in the three HCC cell lines than in L02 cells (Fig. 1C). Using the UALCAN webtool, the methylation levels of the DNA promoter region of HIGD1A were observed to be significantly lower in HCC tissues (*n* = 377) than in normal liver tissue (*n* = 50) (Fig. S1A). Moreover, there was a statistically significant decline in the average methylation level within the promoter region of HIGD1A with increasing tumor grade (Fig. S1B). Additionally, HIGD1A methylation levels were positively correlated with individual cancer stages (Fig. S1C). Finally, in alignment with previous findings, the protein and mRNA levels of HIGD1A in the HCC cell lines were induced under hypoxia or low-glucose condition, but not in L02 cells, and HIF-1α expression was induced by hypoxia in all cell lines (Fig. 1E and F). Collectively, these results suggested that methylation levels of the HIGD1A promoter region are lower in HCC than in normal hepatocytes, and elevated HIGD1A expression in HCC might be associated with aberrant epigenetic regulation.

### HIGD1A knockdown inhibits cell proliferation and promotes hypoxia-induced apoptosis in HCC cells

**Fig. 2:**
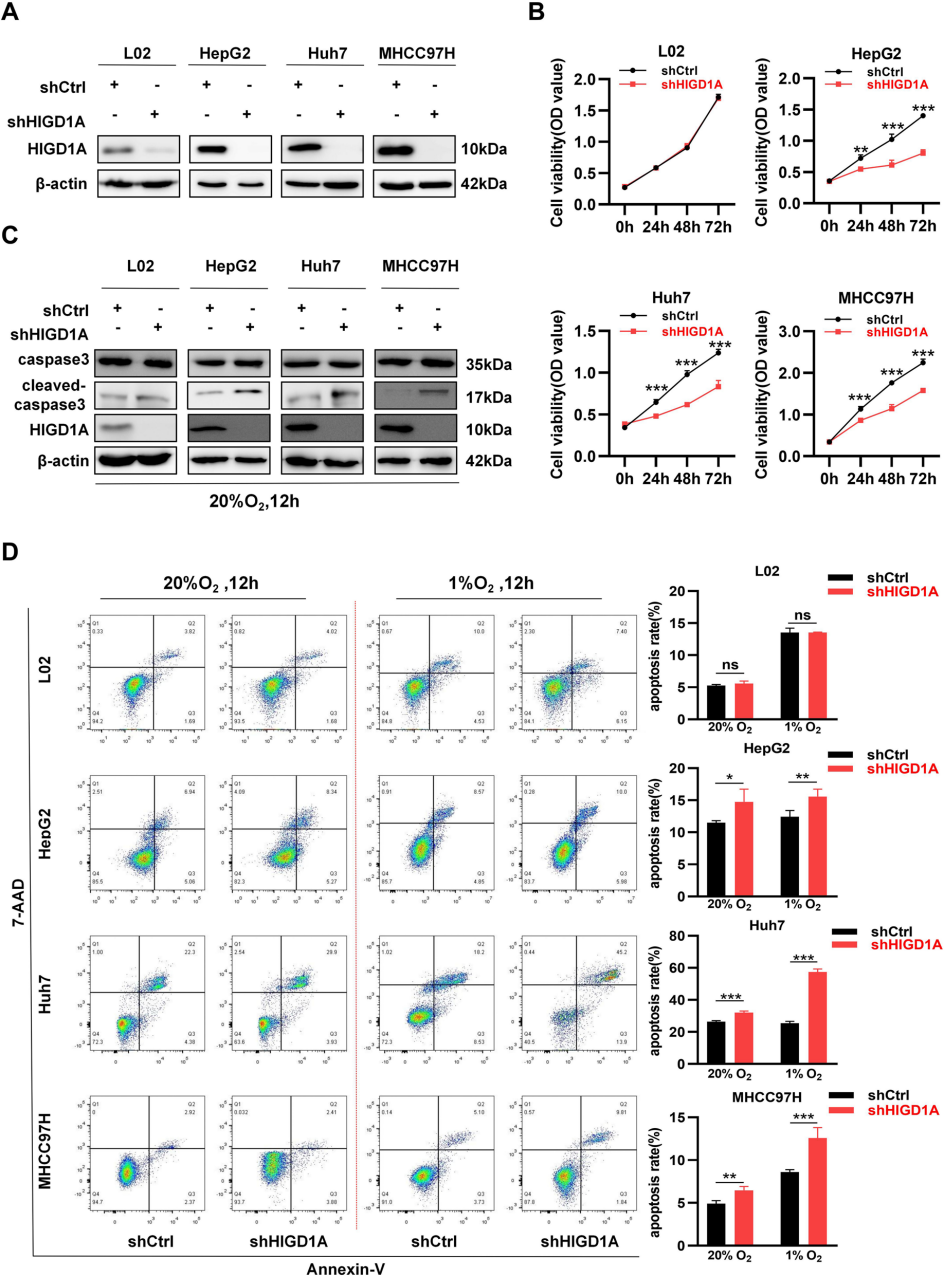
HIGD1A knockdown inhibits cell proliferation and promotes hypoxia-induced apoptosis in HCC cells. **A** Western blotting assay for total HIGD1A protein expression following HIGD1A-knockdown in L02, HepG2, Huh7, and MHCC97H cells. **B** Proliferation ability of L02, HepG2, Huh7, and MHCC97H cells infected with shCtrl or shHIGD1A lentivirus as measured by CCK8 assay at the indicated time points. **C** Protein levels of caspase3 and cleaved-caspase3 were measured in L02, HepG2, Huh7, and MHCC97H cells infected with shCtrl or shHIGD1A lentivirus. **D** Flow cytometry analysis of Annexin V/7-AAD double-stained L02, HepG2, Huh7, and MHCC97H cells infected with shCtrl or shHIGD1A under normoxic or hypoxic condition for 12 h. Left, representative flow cytometric plot. Right, proportions of apoptotic cells. Results shown are mean ± SEM. *P <0.05, **P <0.01, ***P <0.001

Considering HIGD1A was highly expressed in the three HCC cell lines, the loss-of-function experiments were performed by lentivirus-mediated shRNA knockdown of HIGD1A. The knockdown efficiency of shHIGD1A was confirmed in Fig. 2A. First, we examined the cell viability using CCK-8 assay, which revealed that the knockdown of HIGD1A strongly inhibited the proliferation capacity of HCC cell lines while having no discernible impact on L02 cells (Fig. 2B). Then, to determine whether growth inhibition was related to HCC cells apoptosis, we assessed the expression of key apoptosis-related protein cleaved-caspase3 and found that it was elevated in HIGD1A-knockdown HCC cells under normoxic condition (Fig. 2C). Moreover, this elevation was even more pronounced under the hypoxic condition (Fig. S2A). Further detection of apoptosis by flow cytometry analysis yielded similar results, with a higher frequency of Annexin-V positive cells in the hypoxic compared with the normoxic condition. Consistently, HIGD1A knockdown in HCC cell lines resulted in a significant increase of apoptosis cells under hypoxic condition. However, it exerted a minimal impact on the apoptosis of L02 cells (Fig. 2D). Taken together, these findings suggested that HIGD1A knockdown exerts inhibitory effects on cell proliferation while concurrently facilitating hypoxia-induced apoptosis in HCC cells.

### HIGD1A knockdown inhibits the migration and invasion of HCC cells

**Fig. 3:**
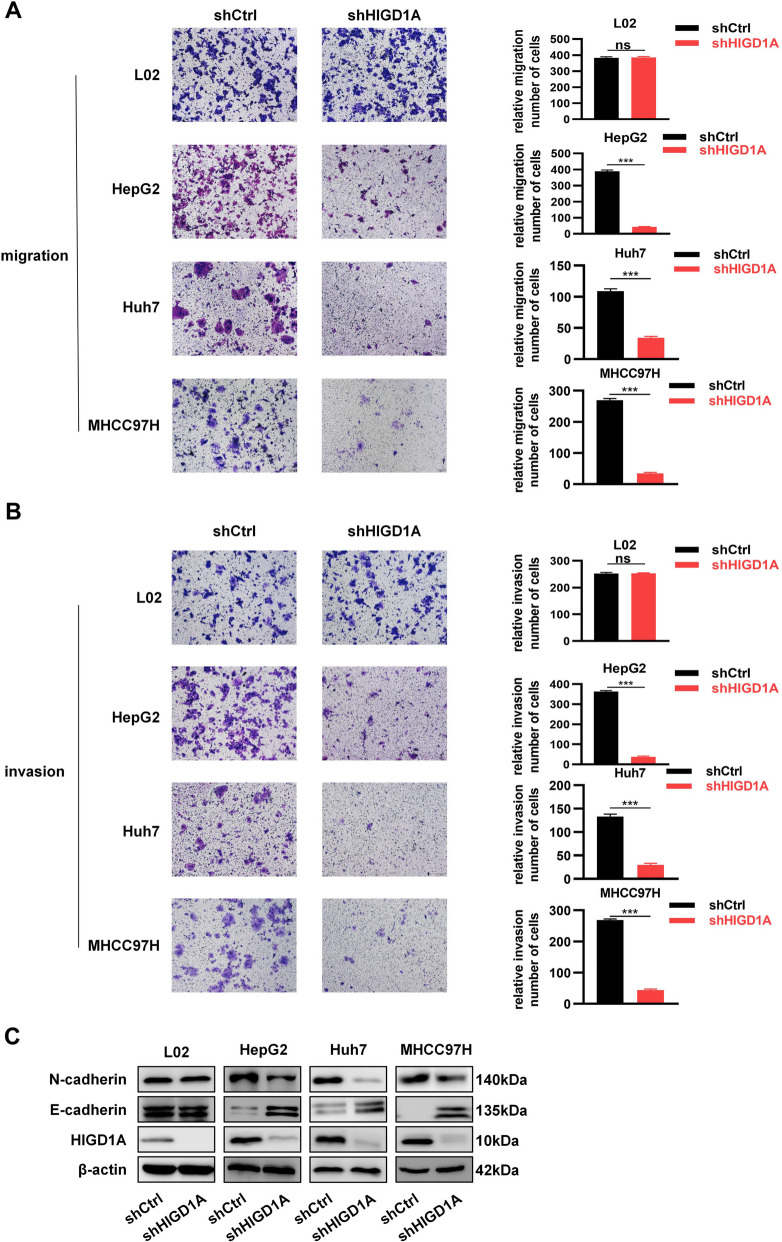
HIGD1A knockdown inhibits the migration and invasion of HCC cells. **A** Cell migration ability of L02, HepG2, Huh7, and MHCC97H cells infected with shCtrl or shHIGD1A lentivirus was analyzed by transwell assay. Representative images of the transwell assay (left) and quantitative data of migrated cells (right) are shown. **B** Cell invasion ability of L02, HepG2, Huh7, and MHCC97H cells infected with shCtrl or shHIGD1A lentivirus as analyzed by transwell assay. Representative images of the transwell assay (left) and quantitative data of migrated cells (right) are shown. **C** Expression of EMT markers E-cadherin and N-cadherin in L02, HepG2, Huh7, and MHCC97H cells infected with shCtrl or shHIGD1A lentivirus as examined by western blotting. Results shown are mean ± SEM. *P <0.05, **P <0.01, ***P <0.001; ns, not significant. Scale bar, 100µm

The Matrigel model was used to evaluate the effects of HIGD1A knockdown on HCC cell migration and invasion function. We found that the knockdown of HIGD1A in all three HCC cell lines significantly decreased the number of migrating cells (Fig. 3A) and invading cells (Fig. 3B). However, it had no significant effect on L02 cells. Furthermore, given that epithelial-to-mesenchymal transition (EMT) is a hallmark of elevated cell mobility and invasion, we tested the expression of several EMT markers. As shown in Fig. 3C, HIGD1A knockdown suppressed mesenchymal marker N-cadherin and upregulated epithelial marker E-cadherin in all three HCC cell lines, but not in L02 cells. Overall, these results demonstrate that HIGD1A overexpression was involved in regulating cell migration and invasion and inducing the EMT process in HCC cells.

### RNA-seq analysis reveals that HIGD1A knockdown causes cell cycle arrest in the G2/M phase through downregulation of c-Myc target genes

**Fig. 4:**
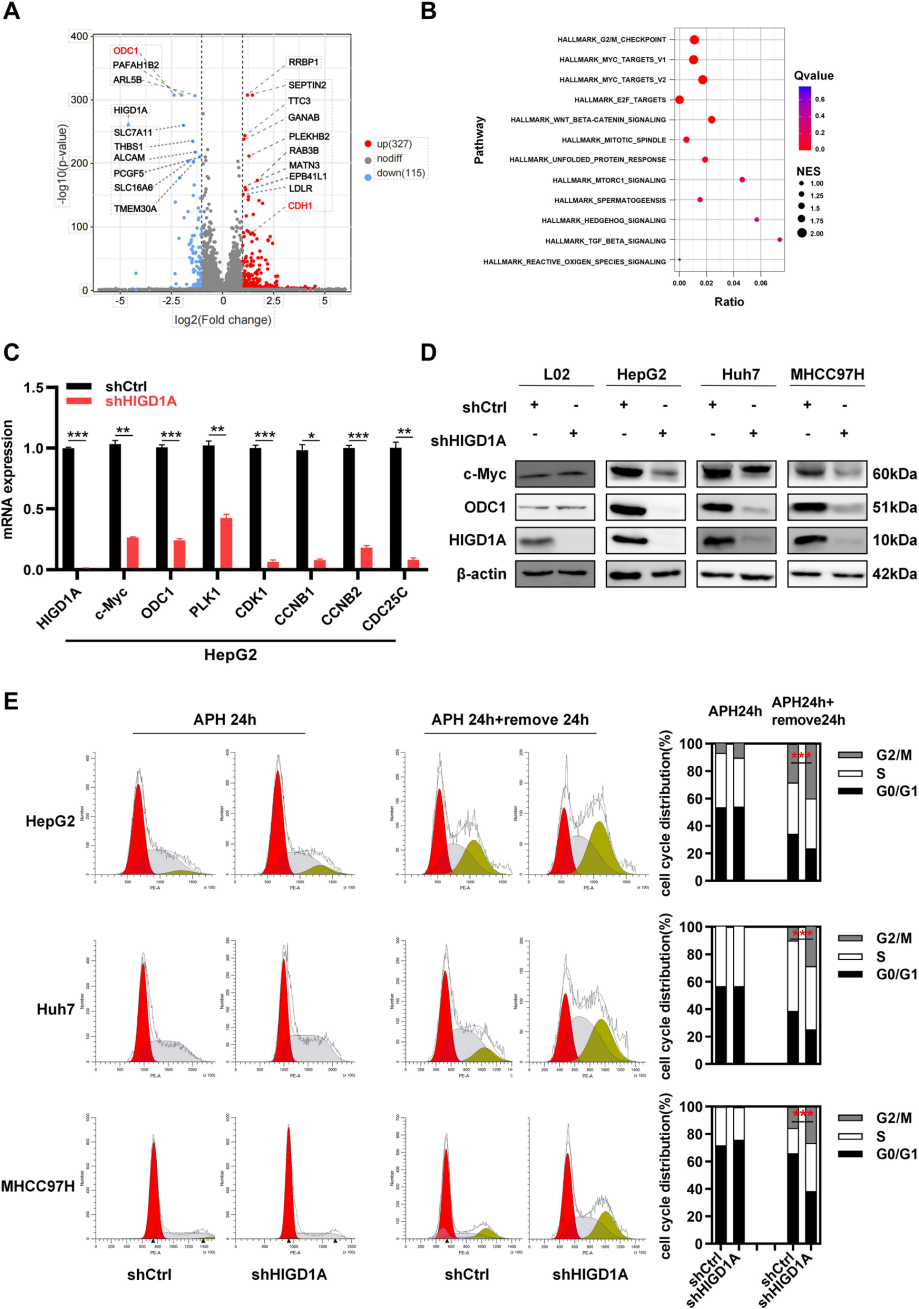
HIGD1A knockdown causes cell cycle arrest in the G2/M phase through downregulating c-Myc target genes. **A** Transcriptome strategy of RNA-seq conducted on HepG2 cells infected with shCtrl or shHIGD1A lentivirus for 48 h. Each group contained three biological replicates. Volcano plots of differential gene expression in shCtrl and shHIGD1A infected HepG2 cells. **B** Signaling pathway enrichment in different groups analyzed by GSEA. Bubble plot shows the main decreased molecular hallmarks (GSEA) enriched in the shHIGD1A groups. Normalized enrichment scores (NES) across gene sets are shown. **C** mRNA levels of HIGD1A, c-Myc, ODC1, and markers of G2/M checkpoints in HepG2 cells infected with shCtrl or shHIGD1A lentivirus. β‐actin was used as a loading control. **D** Protein levels of HIGD1A, c-Myc, and ODC1 in HepG2, Huh7, and MHCC97H cells infected with shCtrl or shHIGD1A lentivirus. **E** Left: HepG2, Huh7, and MHCC97H cells treated with shCtrl or shHIGD1A were synchronized with APH for 24 h. After the removal of APH, HepG2, Huh7, and MHCC97H cells were stained with PI and analyzed by flow assay. Right: proportion of cell populations in G1/S and G2/M phases. Results shown are mean ± SEM. *P <0.05, **P <0.01, ***P <0.001

To elucidate the underlying mechanism of HIGD1A regulation on HCC cancer phenotype, we conducted an RNA-seq analysis on HepG2 cells that had been infected with shCtrl and shHIGD1A. The DEGs were visualized using a volcano plot, and a total of 442 genes (327 upregulated and 115 downregulated genes) exhibited significantly altered mRNA expression in shHIGD1A cells (Fig. 4A). A heatmap depicting the top 10 most significantly upregulated and downregulated genes can be found in Fig. S3A. Among these genes, Ornithine decarboxylase 1 (ODC1), a rate-limiting enzyme required for polyamine biosynthesis, was the most significantly downregulated gene in shHIGD1A cells, whereas CDH1 (encoding E-cadherin), a well-established surface adhesion molecule, was upregulated as mentioned above (Fig. 3C). Furthermore, GSEA analysis revealed that DEGs related gene sets including G2/M checkpoints, MYC targets V1, MYC targets V2, E2F targets, WNT/beta-catenin signaling and mitotic spindle were downregulated in shHIGD1A cells (Fig. 4B, Fig. S3B). In line with the RNA-seq results, we observed that the knockdown of HIGD1A led to the inhibition of protein and mRNA expression of c-Myc and ODC1 in the three HCC cell lines (Figs. 4C, D, and Fig. S3C).

As mentioned above, the gene set associated with G2/M checkpoints was significantly downregulated in the shHIGD1A group. Then we examined the impact of HIGD1A knockdown on cell cycle distribution. The proportion of cells in G0/G1 phase in the shHIGD1A group decreased, whereas the proportion in G2/M phase increased, demonstrating that HIGD1A knockdown led to a significant arrest of HCC cells at G2/M phase. To further confirm these results, cells were synchronized at the G1/S boundary using APH, a DNA polymerase inhibitor. After treatment for 24 h, APH was removed to initiate cell cycle progression, and PI staining was performed 24 h later. As expected, HIGD1A knockdown cells showed a significant G2/M cell cycle arrest with a concomitant reduction in the percentage of cells in the G1 and S phases (Fig. 4E). We also examined the mRNA levels of G2/M checkpoint markers, and found the mRNA expression of PLK1, CDK1, CCNB1, CCNB2, and CDC25C was also significantly decreased in HIGD1A knockdown cells (Fig. 4C and Fig. S3C). Considering ODC1 was a target gene of c-Myc [28], and ODC1 silencing arrested the cell cycle at the G2/M phase [29], we proposed that HIGD1A knockdown inhibited ODC1 expression and arrested cell cycle at the G2/M phase through downregulation of c-Myc.

### HIGD1A transcriptionally activates c-Myc expression to regulate the cancer phenotype of HCC cells

**Fig. 5:**
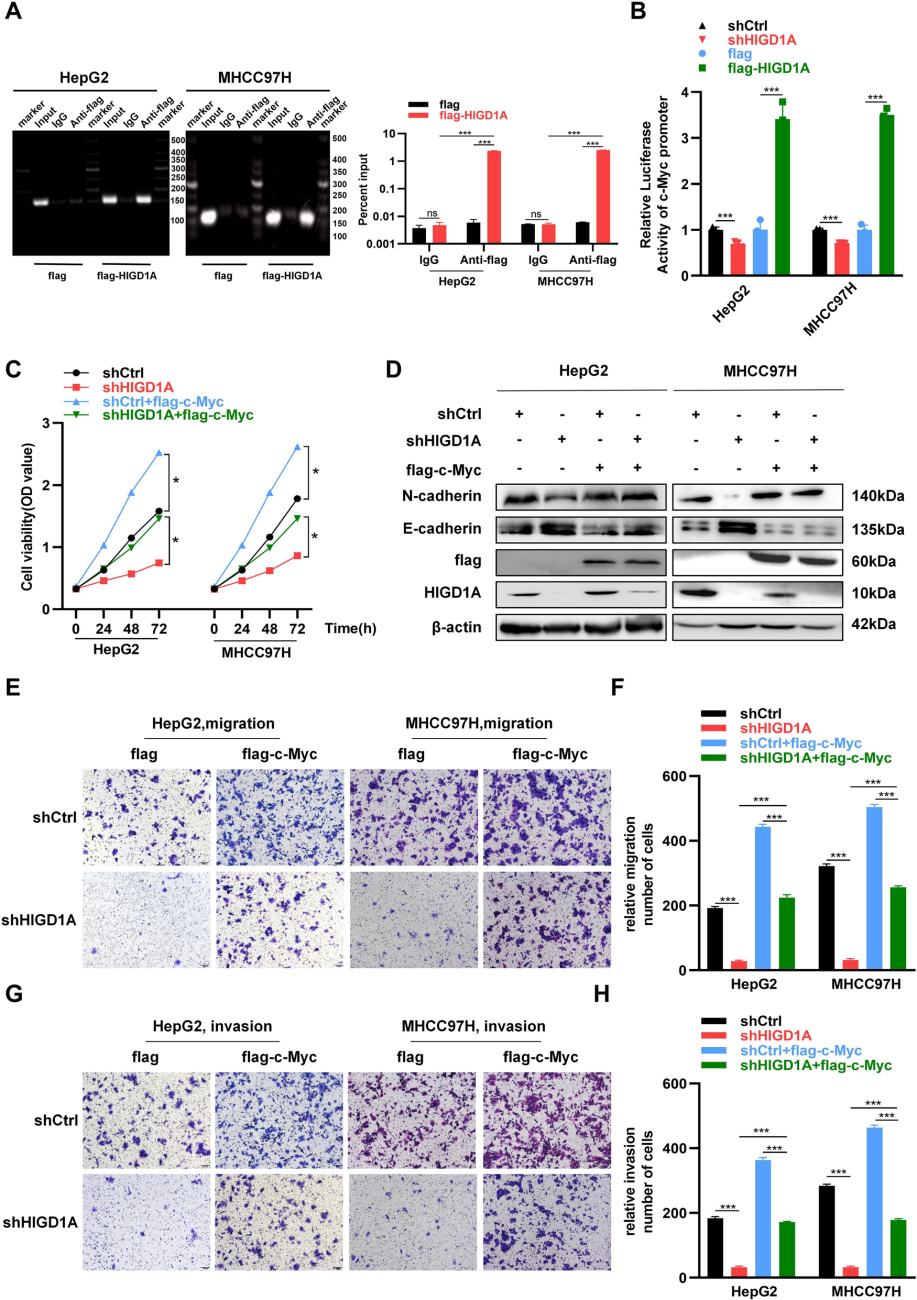
HIGD1A transcriptionally activates c-Myc expression to regulate the cancer phenotype of HCC cells. **A** ChIP assay confirming that c-Myc is transcriptionally activated by HIGD1A. Representative electrophoresis images (left) and RT-PCR (right) results for the ChIP assay. **B** Luciferase reporter assay was performed in HepG2 and MHCC97H cells infected with shCtrl or shHIGD1A lentivirus and transfected with pcDNA3.1 or pcDNA3.1-HIGD1A, respectively. **C** Cell viability of c-Myc-overexpressing HepG2 and MHCC97H cells infected with shCtrl or shHIGD1A lentivirus as measured by CCK8 assay at the indicated time points. **D** Western blotting analysis indicating the expression of c-Myc and EMT markers, E-cadherin and N-cadherin in c-Myc-overexpressing HepG2 and MHCC97H cells infected with shCtrl or shHIGD1A lentivirus. **E** and **F** Transwell assay showing that c-Myc overexpression reversed shHIGD1A-induced inhibition of cell migration in HepG2 and MHCC97H cells. **G** and **H** Transwell assay with Matrigel demonstrated c-Myc overexpression reversed shHIGD1A-induced inhibition of cell invasion in HepG2 and MHCC97H cells. Results shown are mean ± SEM. *P <0.05, **P <0.01, ***P <0.001. Scale bar, 100µm

Given that the knockdown of HIGD1A decreased mRNA levels of c-Myc, suggesting a potential influence on c-Myc transcription, we conducted a ChIP assay and observed the direct binding of HIGD1A to the promoter region of c-Myc (Fig. 5A). Then, we evaluated whether HIGD1A affected c-Myc transcription activity. As expected, the knockdown of HIGD1A in HepG2 and MHCC97H cells led to significantly reduced luciferase activity of the c-Myc promoter compared to the control group. In contrast, the overexpression of HIGD1A induced an increase in luciferase activity (Fig. 5B).

Further, we investigated whether overexpression of c-Myc could reverse the growth inhibition or decreased migratory ability due to HIGD1A knockdown. As expected, our results demonstrated that the overexpression of c-Myc effectively rescued the proliferative capacity of cells subjected to HIGD1A knockdown (Fig. 5C). Also, the overexpression of c-Myc reversed the HIGD1A-knockdown-induced changes in the expression of EMT markers (Fig. 5D). These findings were in line with the outcomes of functional assays assessing migration (Fig. 5E and F) and invasion (Fig. 5G and H) using transwell assays.

### The polyamine metabolism regulated by ODC1 is involved in the modulation of the tumor phenotype by HIGD1A in HCC cells

**Fig. 6:**
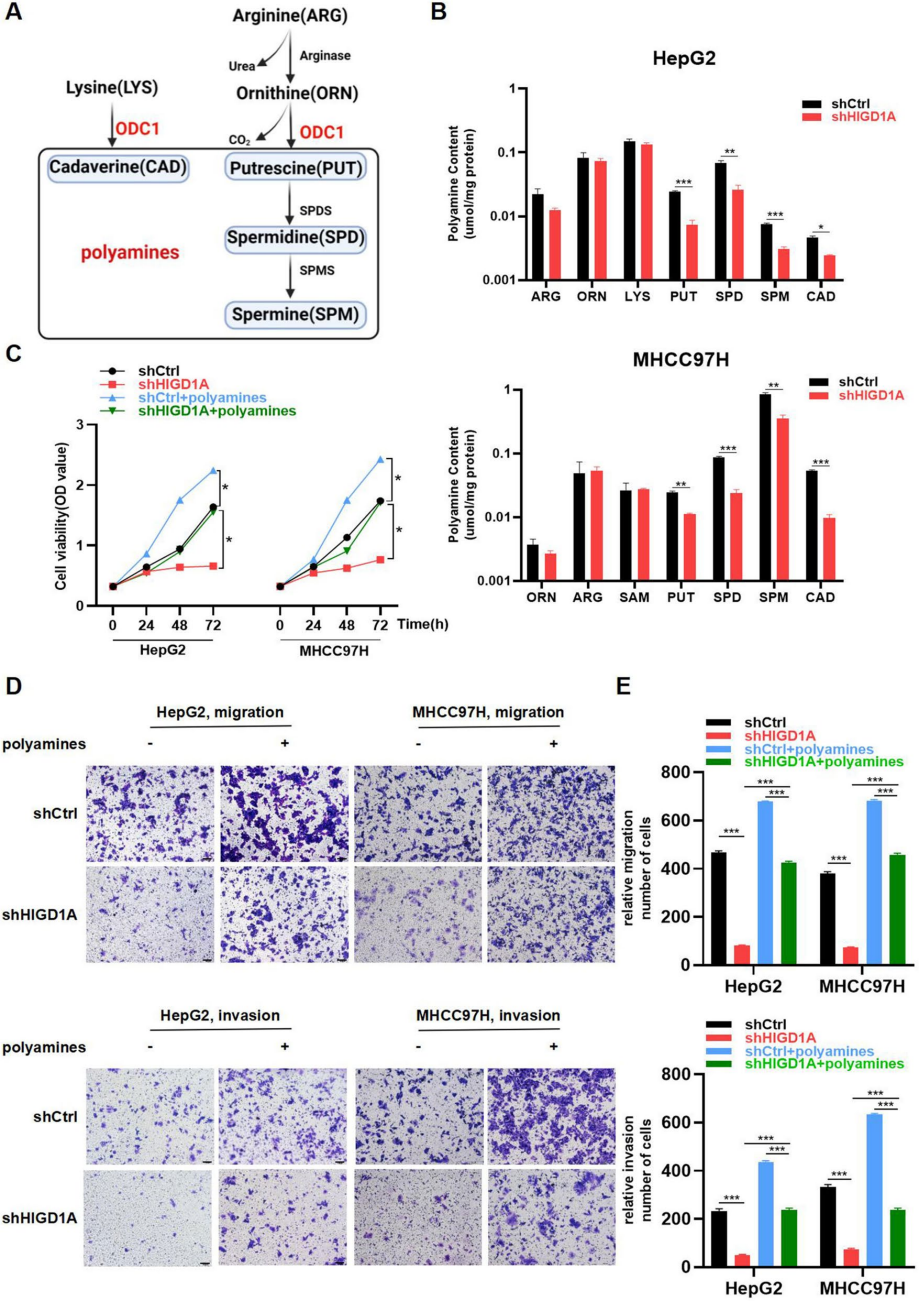
The polyamine metabolism regulated by ODC1 is involved in the modulation of the tumor phenotype by HIGD1A in HCC cells. **A** Simplified scheme of polyamine biosynthesis pathways. SPDS: spermidine synthase; SPMS: spermine synthase. **B** HPLC analysis showing that putrescine, spermidine, spermine, and cadaverine levels were significantly decreased in HepG2 and MHCC97H cells infected with shHIGD1A lentivirus compared with those in the shCtrl group. **C** CCK8 assay showing that exogenous polyamines attenuated shHIGD1A-induced inhibition of cell proliferation in HepG2 and MHCC97H cells. **D** Transwell assay showing that exogenous polyamines restored shHIGD1A-induced inhibition of cell migration in HepG2 and MHCC97H cells. **E** Transwell assay with Matrigel showed that exogenous polyamines restored shHIGD1A-induced inhibition of cell invasion in HepG2 and MHCC97H cells. Results shown are mean ± SEM. *P <0.05, **P <0.01, ***P <0.001. Scale bar, 100µm

ODC1 is the first rate-limiting enzyme involved in the biosynthesis of polyamines [30] such as putrescine (PUT), spermidine (SPD), spermine (SPM), and cadaverine (CAD) within cells, as illustrated in Fig. 6A. Given the essential role of ODC1 in polyamine metabolism, we conducted an HPLC coupled with mass spectrometry analysis of polyamine levels in HepG2 and MHCC97H cells from both the shCtrl and shHIGD1A groups. Notably, the levels of PUT, SPD, SPM, and CAD displayed a significant reduction in the shHIGD1A group (Fig. 6B). Importantly, the amino acids like arginine, ornithine, and lysine remained unchanged following HIGD1A knockdown, suggesting that the reduction in polyamine levels was specifically attributed to the modulation of ODC1 expression.

To discern the implications of altered polyamine levels on cell phenotype, we subjected HIGD1A-knockdown HepG2 and MHCC97H cells to treatment with a polyamine mixture for 3 days. Strikingly, the addition of exogenous polyamines effectively reversed the inhibitory effects of shHIGD1A on cell growth (Fig. 6C). Moreover, polyamine supplementation significantly counteracted the reductions induced by shHIGD1A in the migration and invasion capabilities of cells as observed in Matrigel assays (Fig. 6D-G). Taken together, these results suggested that the knockdown of HIGD1A inhibited the expression of ODC1, leading to a decrease in the levels of polyamine metabolites, and resulted in the suppression of HCC cell proliferation and invasion.

### HIGD1A knockdown targets c-Myc–ODC1 nexus to inhibit HCC tumor growth in vivo model

**Fig. 7:**
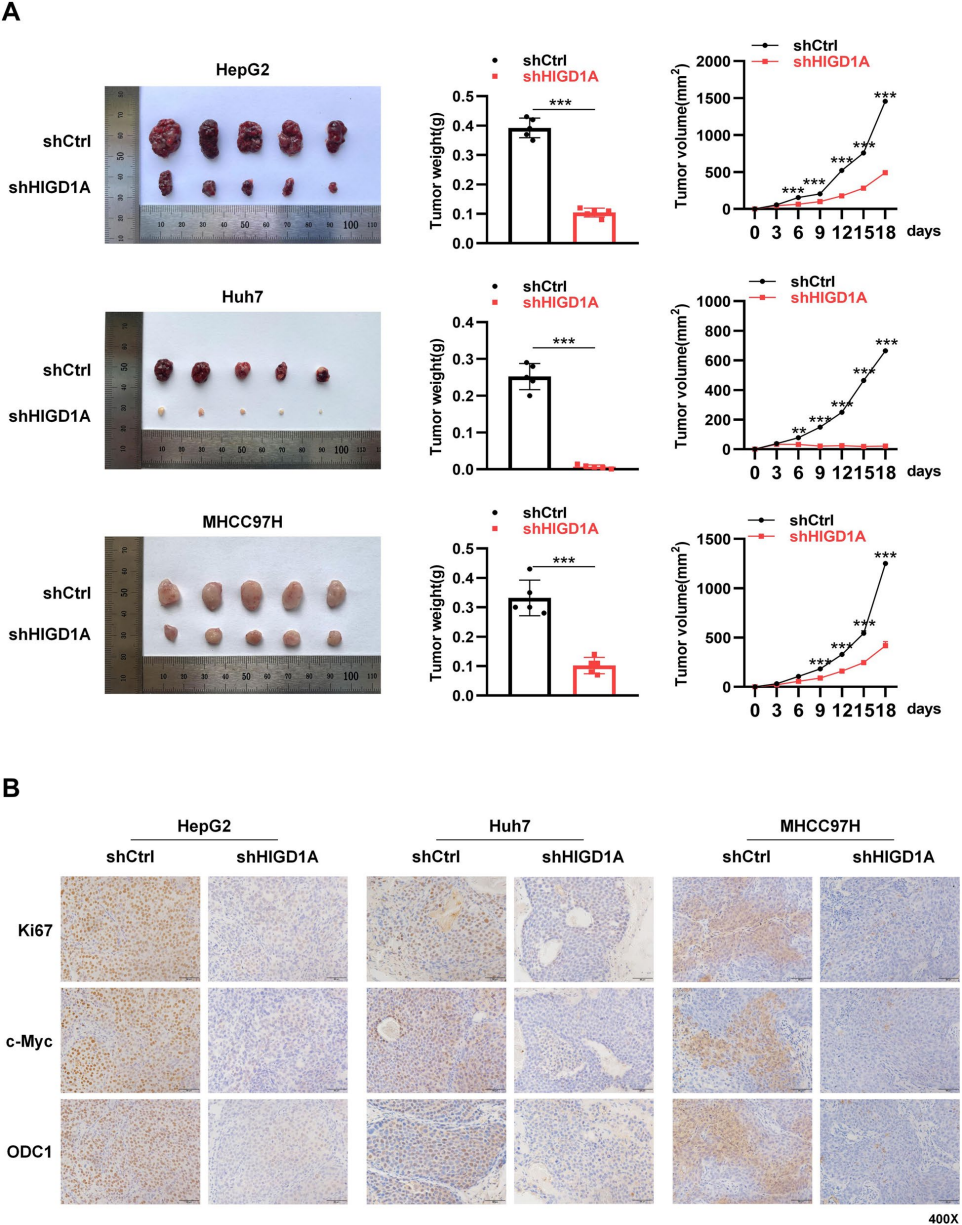
HIGD1A knockdown targets c-Myc–ODC1 nexus to inhibit HCC tumor growth in xenografted nude mice model. **A** Images of HepG2, Huh7, and MHCC97H xenograft tumors in nude mice of the shCtrl group and shHIGD1A group (*n* = 5 mice per group). The middle and right graphs show the final weights of the tumors and the tumor growth curve, respectively. **B** Immunohistochemical staining of Ki67, c-Myc, and ODC1 in HepG2, Huh7, and MHCC97H subcutaneous xenograft tumor tissues from the shCtrl group and shHIGD1A group. Results shown are mean ± SEM. *P <0.05, **P <0.01, ***P <0.001. Scale bar, 50µm

Finally, we investigated the impact of HIGD1A on HCC tumor growth using subcutaneous and intrahepatic orthotopic xenograft tumor models established with three different HCC cell lines. The results demonstrated that the knockdown of HIGD1A significantly influenced tumor growth across all three HCC cell lines, with a significant reduction in both volume and weight of subcutaneous tumors (Fig. 7A) and orthotopic tumors (Fig. S4A) in the HIGD1A knockdown groups compared to the control group. Moreover, immunohistochemical analysis of Ki67, c-Myc, and ODC1 expression was conducted in subcutaneous xenograft tumor models. Consistent with the *in vitro* findings, the expression of Ki67 was markedly reduced in the HIGD1A knockdown groups of different HCC cell lines, indicating a suppressed tumor proliferation. Furthermore, the expression levels of the HIGD1A-regulated target genes, c-Myc and ODC1, were also significantly inhibited (Fig. 7B). These findings further validated that HIGD1A exerted an oncogenic effect by modulating the tumor phenotype of HCC cells through the targeting of the c-Myc and ODC1 genes.

## Discussion

**Fig. 8:**
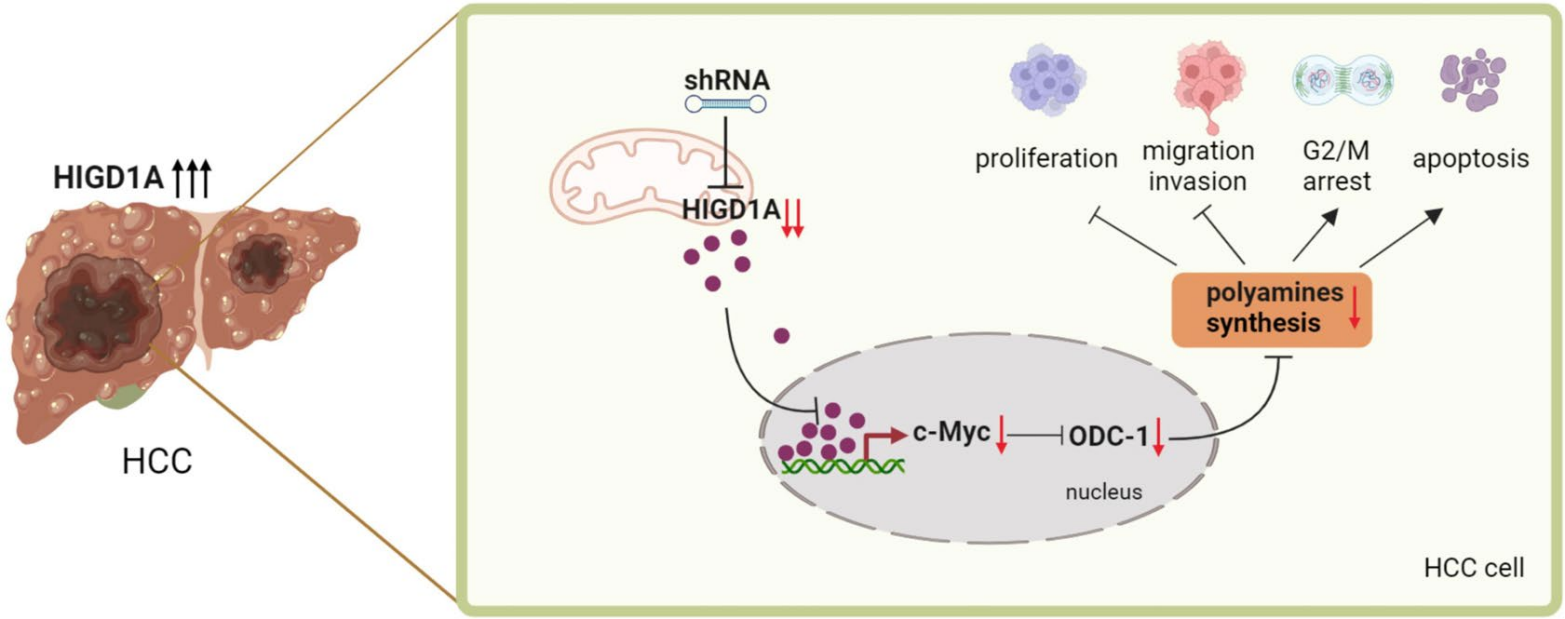
Schematic model of the mechanism of HIGD1A action on HCC phenotype. HIGD1A is overexpressed in HCC and knockdown of HIGD1A inhibits the HCC cancer phenotype by regulating polyamine metabolism through the c-Myc–ODC1 nexus. This image was created with BioRender.com

In this study, we demonstrated that the expression of the HIGD1A gene was upregulated in both HCC tissues and cell lines. By silencing the expression of the HIGD1A gene, we observed the cell cycle arrest at the G2/M phase and increased apoptosis under hypoxic conditions, then resulted in the inhibition of proliferation, migration, and invasion capabilities in HCC cells. Further exploration of the mechanism revealed that HIGD1A could directly activate the transcription of the c-Myc gene, thereby activating the expression of its downstream target gene ODC1. This regulatory effect influenced polyamine metabolism, thereby affecting the phenotypic characteristics of HCC cells (Fig. 8). These results indicated that increased expression of HIGD1A contributed to the growth and metastasis of HCC and could potentially serve as a therapeutic target for liver cancer.

In agreement with our study, H.J. An et al. found that upregulation of HIGD1A promoted tumor cell proliferation, migration, and invasion in pancreatic cancer cells. However, HIGD1A knockdown in pancreatic cancer cells did not affect cell apoptosis instead of causing cell cycle retardation through induction of p27^KIP1^ and RB hypo-phosphorylation [19]. In addition, HIGD1A was found to be upregulated in glioma cells and the knockout of HIGD1A led to decreased cell proliferation and increased apoptosis in glioma [31]. In contrast, HIGD1A was found to be underexpressed in colon adenocarcinoma cells, and its overexpression impaired the proliferation, migration, and invasiveness of colon adenocarcinoma cells [20], indicating that HIGD1A plays a pro-oncogenic or tumor-suppressive role relating with the genetic background of the tumor.

Of interest, we observed that the knockdown of HIGD1A significantly inhibits proliferation, migration, and invasion of HCC cells, but with limited effects on normal liver cells L02. There were two potential explanations for this discrepancy. Firstly, HIGD1A is underexpressed in L02 cells as a result of HIGD1A promoter hypermethylation. As a consequence, the impact of HIGD1A knockdown on cell phenotype in L02 cells was relatively modest. Secondly, using immunofluorescence staining, we found that HIGD1A primarily localized to the nucleus in HCC cells, while in L02 cells, it's predominantly found in the cytoplasm (Fig. S5A). This differential subcellular distribution between the nucleus and cytoplasm suggested that HIGD1A primarily exerted its effects by translocating to the nucleus to activate target genes, consequently explaining the substantial effects of HIGD1A knockdown on HCC cell phenotype.

It has been reported that HIGD1A interacts with the electron transport chain, leading to

the activation of mitochondrial ROS-dependent AMPK and subsequent reduction in respiration and total ROS levels [16]. This orchestrated process promotes tumor cell survival and suppresses growth, implying its crucial role in maintaining mitochondrial homeostasis. The HIG1 protein family has two mammalian homologs, namely HIGD1A and HIGD2A, are both induced by HIF-1 [32]. Our recent study has revealed that silencing HIGD2A inhibits the proliferation and migration of HCC cells by suppressing mitochondrial respiratory chain function [33]. In light of these findings, we sought to explore whether HIGD1A knockdown is associated with mitochondrial oxidative phosphorylation function. However, our findings revealed that HIGD1A knockdown did not elicit significant changes in oxygen consumption rate (OCR) or extracellular acidification rate (ECAR) compared to control cells (Fig. S6A and B). One possible explanation is that HIGD1A and HIGD2A exhibit independent and overlapping functions in the biogenesis of respiratory complexes, allowing HIGD2A to partially substitute for HIGD1A's function [34]. Certainly, further experimental validation is needed to confirm this hypothesis.

Polyamines were essential for cell proliferation and survival, and both the growth of tumor cells and tumor progression require elevated levels of polyamines [35]. Inhibiting polyamine metabolic processes or reducing polyamine metabolic products can exert anti-tumor effects [36]. ODC1 is a rate-limiting enzyme in the downstream polyamine biosynthetic pathway [37]. In liver cancer, the expression and activity of ODC1 were elevated compared to normal tissues [29]. Difluoromethylornithine is a highly effective and specific inhibitor of ODC1 [38] and its treatment resulted in intracellular polyamine depletion, cell cycle arrest [39], thereby inhibiting tumor cell growth. Considering the significant decrease in the expression of c-Myc and ODC1 following HIGD1A silencing in HCC cells, it suggested that the intracellular polyamine metabolic pathway was inhibited, leading to a reduction in polyamine products. This inhibition consequently suppressed HCC cell proliferation and other phenotypes. Based on the above observation, we speculated that the polyamine metabolic products might serve as the effector molecules through which HIGD1A regulated the phenotypes of HCC cells. Therefore, our results further confirmed the proposed strategy that targets c-Myc and ODC1 in HCC treatment.

## Conclusions

Current anti-tumor therapies for HCC remain unsatisfactory, predominantly due to their limited efficacy in blocking the high rates of recurrence and metastasis. Our study has revealed that targeted modulation of HIGD1A yields a notable inhibition to the proliferative, migratory, and invasive capacities of HCC cells. This effect is associated with polyamine levels regulated by the c-Myc–ODC1 interplay. Importantly, our study reveals the discrepancies in HIGD1A expressions and functionalities between HCC cells and their normal counterparts. This discernment helps us to identify the underlying mechanisms driving HCC tumorigenesis. Moreover, it implies a promising target for HCC therapeutic intervention, suggesting that the development of HIGD1A inhibitors could be potentially used for the treatment of liver cancer.

### Supplementary Information

**Supplementary Material 1.** 

**Supplementary Material 2.** 

**Supplementary Material 3.** 

## Data Availability

All data needed to evaluate the conclusions in the paper are present in the paper and/or the Supplementary Materials. Additional data related to this paper may be requested from the corresponding authors.

## Abbreviations

HCC: Hepatocellular carcinoma
HIGD1A: Hypoxia-inducible gene domain protein-1a
ODC1: Ornithine decarboxylase 1
HIFs: Hypoxia-inducible factors
CHIP: Chromatin immunoprecipitation
HPLC: High-Performance Liquid Chromatography
DNMT1: DNA methyl transferase 1
CHIP: Chromatin immunoprecipitation
APH: Aphidicolin
OCR: Oxygen Consumption Rate
ECAR: Extracellular Acidification Rate
EMT: Epithelial-to-mesenchymal transition
GSEA: Gene set enrichment analysis
DEGs: Differentially expressed genes
