# Supplementary material for: Elevated expression of HIGD1A drives hepatocellular carcinoma progression by regulating polyamine metabolism through c-Myc–ODC1 nexus
Source: Cancer Metab. 2024 Feb 23;12:7. doi: 10.1186/s40170-024-00334-6 (PMC10893642; doi:10.1186/s40170-024-00334-6)
Supplement: Supplementary file 1 — Supplementary Material 1. [file 40170_2024_334_MOESM1_ESM.docx]

Supplementary information

**Elevated expression of HIGD1A drives hepatocellular carcinoma progression by regulating polyamine metabolism through c-Myc–ODC1 nexus**

Haixing Zhang ^1#^, Xiaoran Li^1#^, Ziying Liu^1#^, Zimo Lin^1^, Kuiyuan Huang^1^, Yiran Wang^1^, Yu Chen^1^, Leyi Liao^2^, Leyuan Wu^1^, Zhanglian Xie^1^, Jinlin Hou^1🖂^, Xiaoyong Zhang^1🖂^, Hongyan Liu^1🖂^

^1^ State Key Laboratory of Organ Failure Research, Guangdong Provincial Key Laboratory of Viral Hepatitis Research, Department of Infectious Diseases, Nanfang Hospital, Southern Medical University, Guangzhou, China;

^2^ Division of Hepatobiliopancreatic Surgery, Department of General Surgery, Nanfang Hospital, Southern Medical University, Guangzhou, China.

^#^ These authors contributed equally to this work.

^🖂^Corresponding authors：

Hongyan Liu, MD

Hepatology Unit and Department of Infectious Diseases,

Nanfang Hospital, Southern Medical University,

1838 Guangzhou Avenue North, Guangzhou, 510515, China

E-mail: liuhongyan123@smu.edu.cn

Phone: +86-20-62787430

Xiaoyong Zhang, PhD

Hepatology Unit and Department of Infectious Diseases,

Nanfang Hospital, Southern Medical University,

1838 Guangzhou Avenue North, Guangzhou, 510515, China

E-mail: xiaoyzhang@smu.edu.cn

Phone: +86-20-62787830

Jinlin Hou, Professor

Hepatology Unit and Department of Infectious Diseases,

Nanfang Hospital, Southern Medical University,

1838 Guangzhou Avenue North, Guangzhou, 510515, China

E-mail: jlhousmu@163.com

Phone: +86-20-61641941

**Cover letter**

September 1, 2023

Dear Editor,

Please find our manuscript entitled “Elevated expression of HIGD1A drives hepatocellular carcinoma progression by regulating polyamine metabolism through c-Myc–ODC1 nexus” for consideration for publication as an original article in the Cancer Metabolism.

Our study focuses on the overexpression of Hypoxia-induced gene domain protein-1a (HIGD1A) in hepatocellular carcinoma (HCC), which is induced by hypoxia or low-glucose condition. Through a series of loss-of-function experiments, we verified that HIGD1A knockdown in HCC cells arrests the cell cycle at the G2/M phase and promotes hypoxia-induced cell apoptosis, resulting in significant inhibition of cell proliferation, migration, and invasion. The underlying mechanism behind these effects is the decline of polyamine levels through inhibiting the c-Myc–ODC1 nexus. In summary, our study establishes a link between HIGD1A and HCC through the alteration of polyamine metabolism, highlighting the role of HIGD1A as an oncogene and suggesting its potential as a promising therapeutic strategy for treating HCC. We hope that the findings of this manuscript align well with the scopes and requirements of this journal.

This manuscript has not been published and is not under consideration for publication elsewhere. We have no conflicts of interest to disclose. All authors have read and approved the final version of the manuscript. Thank you for your consideration.

Sincerely,

Hongyan Liu, Associate Professor

Department of Infectious Diseases,

Nanfang Hospital, Southern Medical University, Guangzhou, China

1838 Guangzhou Avenue North, Guangzhou, 510515, China

E-mail address: liuhongyan123@smu.edu.cn

Xiaoyong Zhang, Professor

Hepatology Unit and Department of Infectious Diseases,

Nanfang Hospital, Southern Medical University,

1838 Guangzhou Avenue North, Guangzhou, 510515, China

E-mail: xiaoyzhang@smu.edu.cn

Jinlin Hou, Professor

Hepatology Unit and Department of Infectious Diseases,

Nanfang Hospital, Southern Medical University,

1838 Guangzhou Avenue North, Guangzhou, 510515, China

E-mail: jlhousmu@163.com

**Methods**

**Seahorse XF cell Mito Stress Test**

Seahorse experiments were performed on a Seahorse XFe96 Analyzer. The Seahorse XF cell Mito Stress Test kit (Agilent, USA) was used to examine the rate of oxygen consumption (OCR) in accordance with the instructions provided by the manufacturer. In short, HepG2 cells were infected with either the lentivirus shHIGD1A or shCtrl and then placed onto Seahorse cell culture microplates at a density of 7200 cells per well. The Seahorse XFe96 extracellular flux analyzer was used to determine the OCR after a period of over 24 h. Agilent's Mito Stress test involved taking four initial measurements before introducing electron transport chain inhibitors, namely 1.5 µM oligomycin, 1.0 µM FCCP, or 0.5 µM rotenone/antimycin. OCR data were normalized to call count. Measurement was conducted on basal respiration and respiration capacity. Results were analyzed in WAVE software.

**Seahorse XF glycolytic rate assay**

In the Seahorse XF Glycolysis rate assay, glycolysis capacity after glucose deprivation was examined. In short, HepG2 cells that were infected with lentivirus shHIGD1A or shCtrl were seeded according to the instructions for the Mito Stress Test. In port A, a concentration of 0.5 µM of Rotenone/Antimycin was introduced, followed by the injection of 62 µl of the glycolysis inhibitor, 2-deoxy-D-glucose (2-DG), at a concentration of 50 mM into port B. This was done to bind to hexokinase and hinder glucose metabolism. During the assay, the rate of proton efflux in glycolysis, known as the extracellular acidification rate (ECAR), was determined.

**Immunofluorescence staining**

The cells were placed in a confocal microscopy plate and allowed to grow for 24 h; then, the cultured cells were washed three times with PBS. Following treatment with 4% paraformaldehyde for 20 min, the cells underwent another two rounds of PBS washing and treated with 0.5% Triton-X100 for 30 min to make them permeable. Following another three washes with PBS, the cells were then treated with a PBS solution containing 5% bovine serum albumin at room temperature for 1 h. Subsequently, they were incubated with the HIGD1A antibody (21749-1-AP, Proteintech) overnight at 4 °C. Following thorough washing, the cells were then exposed to the secondary antibody for 1 h at room temperature in a dark environment, rinsed three times with PBS, and subsequently exposed to DAPI (Abcam, USA) for 5 min nuclear staining. Afterwards, the cells were imaged using a ZEISS LSM 880 confocal microscope (Carl Zeiss, USA).

Supplementary Table 1. The lists of the primers.

| shCtrl | TTCTCCGAACGTGTCACGT |
| --- | --- |
| shHIGD1A | AATTGTTGCATATGGATTA |
| HIGD1A Forward | AAGAGGCACCATTCGTACCC |
| HIGD1A Reverse | ACCAACAGTCATTGCTCCTACA |
| GAPDH Forward | GGTGGTCTCCTCTGACTTCAACA |
| GAPDH Reverse | GTTGCTGTAGCCAAATTCGTTGT |
| c-Myc Forward | TCCCTCCACTCGGAAGGAC |
| c-Myc Reverse | TTGGACGGACAGGATGTATGC |
| ODC1 Forward | TTTACTGCCAAGGACATTCTGG |
| ODC1 Reverse | GGAGAGCTTTTAACCACCTCAG |
| PLK1 Forward | AGCCCCTCACAGTCCTCAATAA |
| PLK1 Reverse | TCGACCACCTCACCTGTCTCT |
| CDK1 Forward | AAACTACAGGTCAAGTGGTAGCC |
| CDK1 Reverse | TCCTGCATAAGCACATCCTGA |
| CCNB1 Forward | AAGTCATGGAGAATCTGCTGCAT |
| CCNB1 Reverse | TGGCAGCAATCACAAGAAGAA |
| CCNB2 Forward | CTGTACATGTGCGTTGGCATT |
| CCNB2 Reverse | AAGCCAAGAGCAGAGCAGTAATC |
| CDC25C Forward | GCGGCTACAGAGACTTCTTTCC |
| CDC25C Reverse | CACCTCAGCAACTCAGTCTTGTG |
| h-c-Myc-promoter-Forward | GCTGCAAACTCAACGGGTAA |
| h-c-Myc-promoter-Reverse | CTCATCCTTGGTCCCTCACC |

.

**Fig. S1** **HIGD1A promoter methylation levels are lower in HCC tumor.** **(A)** Comparisons of HIGD1A promoter methylation levels in 377 cases of HCC tumor and 50 cases of adjacent non‐tumor tissues. **(B)** Methylation levels of HIGD1A in HCC patients with different tumor grades. **(C)** Methylation levels of HIGD1A in HCC patients with different individual cancer stages. Results shown are mean ± SEM. *^*^P* <0.05, *^**^P* <0.01, ***P <0.001.

**Fig. S2 HIGD1A knockdown promotes hypoxia-induced apoptosis in HCC cells. (A)** Protein levels of caspase3 and cleaved-caspase3 were measured in L02, HepG2, Huh7, and MHCC97H cells infected with shCtrl or shHIGD1A lentivirus under hypoxic condition 12 h.

**Fig. S3 HIGD1A knockdown causes cell cycle arrest in the G2/M phase through downregulating c-Myc target genes. (A)** Heatmap of differential gene expression in shCtrl and shHIGD1A infected HepG2 cells. **(B)** GSEA was used to analyze the signaling pathways enrichment in shHIGD1A groups. NES, Normalized enrichment

score; FDR, False discovery rate. **(C)** mRNA levels of HIGD1A, c-Myc and ODC1 and

the markers of G2/M checkpoints in Huh7 and MHCC97H cells infected with shCtrl or shHIGD1A lentivirus. β‐actin was used as a loading control. Results shown are mean ± SEM. *^*^P* <0.05, *^**^P* <0.01, ***P <0.001.

**Fig. S4 HIGD1A knockdown inhibits HCC tumor growth in** **liver tumor orthotopic nude mouse model. (A)** **Images of HepG2, Huh7, and MHCC97H orthotopic tumors** **in nude mice of the shCtrl group and shHIGD1A group (n = 4 mice per group). The right graphs show the final weights of the tumors.** **Results shown are mean ± SEM. *P <0.05, **P <0.01, ***P <0.001.**

**Fig. S5 HIGD1A is located in the nucleus of HCC cells. (A)** Confocal immunofluorescence microscopy. L02, HepG2, Huh7, and MHCC97H cells were stained with anti-HIGD1A antibody and Alexa Fluor 488-labeled anti-rabbit IgG Antibody. Scale bar, 20µm.

**Fig. S6 Knockdown of HIGD1A does not affect the mitochondrial function or glycolytic function of HepG2 cells. (A)** OCR in HepG2 cells transfected with shCtrl or shHIGD1A was measured by seahorse analyzer. Right, basal respiration rate and maximal respiration capacity are shown. Rot/AA, rotenone/antimycin A. **(B)** The ECAR in HepG2 cells transfected with shCtrl or shHIGD1A was measured in an XFe96 analyzer after injection of Rot/AA and 2-deoxy-d-glucose (2-DG). Graphical analysis of Basal Glycolysis and Compensatory Glycolysis. Results shown are mean ± SEM.
